# Tomato *ABSCISIC ACID STRESS RIPENING* (*ASR*) Gene Family Revisited

**DOI:** 10.1371/journal.pone.0107117

**Published:** 2014-10-13

**Authors:** Ido Golan, Pia Guadalupe Dominguez, Zvia Konrad, Doron Shkolnik-Inbar, Fernando Carrari, Dudy Bar-Zvi

**Affiliations:** 1 Department of Life Sciences and Doris and Bertie Black Center for Bioenergetics in Life Sciences, Ben-Gurion University of the Negev, Beer-Sheva, Israel; 2 Instituto de Biotecnología, Instituto Nacional de Tecnología Agropecuaria, Buenos Aires, Argentina; ISA, Portugal

## Abstract

Tomato *ABSCISIC ACID RIPENING 1* (*ASR1*) was the first cloned plant *ASR* gene. *ASR* orthologs were then cloned from a large number of monocot, dicot and gymnosperm plants, where they are mostly involved in response to abiotic (drought and salinity) stress and fruit ripening. The tomato genome encodes five *ASR* genes*: ASR1, 2, 3* and *5* encode low-molecular-weight proteins (ca. 110 amino acid residues each), whereas *ASR4* encodes a 297-residue polypeptide. Information on the expression of the tomato *ASR* gene family is scarce. We used quantitative RT-PCR to assay the expression of this gene family in plant development and in response to salt and osmotic stresses. *ASR1* and *ASR4* were the main expressed genes in all tested organs and conditions, whereas *ASR2* and *ASR3/5* expression was two to three orders of magnitude lower (with the exception of cotyledons). *ASR1* is expressed in all plant tissues tested whereas ASR4 expression is limited to photosynthetic organs and stamens. Essentially, *ASR1* accounted for most of *ASR* gene expression in roots, stems and fruits at all developmental stages, whereas *ASR4* was the major gene expressed in cotyledons and young and fully developed leaves. Both *ASR1* and *ASR4* were expressed in flower organs, with *ASR1* expression dominating in stamens and pistils, *ASR4* in sepals and petals. Steady-state levels of *ASR1* and *ASR4* were upregulated in plant vegetative organs following exposure to salt stress, osmotic stress or the plant abiotic stress hormone abscisic acid (ABA). Tomato plants overexpressing *ASR1* displayed enhanced survival rates under conditions of water stress, whereas *ASR1*-antisense plants displayed marginal hypersensitivity to water withholding.

## Introduction

The first member of the tomato *ABSCISIC ACID STRESS RIPENING* (*ASR*) gene family, *ASR1*, was identified by screening a tomato fruit cDNA library with cDNA from stressed leaves [1], hence its name. Since then, a large number of *ASR* orthologs have been cloned from many plant species, including gymnosperms and angiosperms (reviewed by [2]). *ASR* gene families are found in the genomes of both monocots and dicots, but they are missing in the model plant *Arabidopsis* [2]. Interestingly, no *ASR* orthologs have been found in organisms outside the plant kingdom. *ASR* genes have been shown to be induced by abscisic acid (ABA) and abiotic stress, mainly salinity and drought [1], [3]–[18]. They are also highly expressed in ripening fruit [1], [4], [14], [19]–[23], and during potato-tuber development [24], [25].

The tomato ASR gene family consists of five genes localized in one cluster on chromosome 4. Four members of the *ASR* gene family have been cloned from tomato [4], [26]–[30]. These genes encode highly homologous proteins and possess a single intron of different size, but conserved location [4], [27], [28]. Upon completion of the tomato genome sequence [31], a fifth *ASR* gene was annotated, whose exon nucleotide sequence is highly similar to that of *ASR3* (88% identity in coding sequences, see also [32]). The loci of *ASR1–ASR5* genes in the tomato genome are Solyc04g071610, Solyc04g071580, Solyc04g071590, Solyc04g071620 and Solyc04g071600, respectively. Four of the genes (*ASR1–ASR3, ASR5*) encode low-molecular-weight proteins, whereas the polypeptide encoded by *ASR4* is approximately double the size of the other proteins [30]. Wild tomato species also encode this five member ASR gene family. suggesting that this family was not lost during tomato domestication and breeding [30], [33]. Furtheremore, *ASR1, 2* and *4* genes from wild tomato species were also induced by drought and cold. The majority of plant ASR genes encode low molecular weight proteins. In addition, genomes of a number of plant species contain in addition a gene encoding higher molecular ASR polypeptides [25], [34], [35].

ASR proteins have been proposed to belong to the hydrophylin group of proteins [36]. Tomato ASR1 was shown to be a natively unordered protein [37] that possesses chaperone-like activity [38], and was localized to both the cytosol and nucleus [39]. Dual subcellular localization was also shown for the lily pollen ASR protein [40]. Rice ASR1 was also shown to possess chaperone-like activity [41], [42]. ASR1 has Zn^2+^-dependent DNA-binding activity [39], [43]. Upon binding of Zn^2+^, ASR1 becomes structured and dimerizes [26], [37], and is translocated to the nucleus [37], [44], [45]. Zn^2+^ and Fe^3+^ ions affected the structure of a soybean ASR [46]. Nuclear ASR proteins modulate gene expression via binding to specific promoter sequences [14], [39], [47], [48].

*ASR* genes have been shown to play a central role in drought and salinity stress. Overexpression of *ASR* genes resulted in increased tolerance of the transgenic plants to water/osmotic [42], [48]–[51], salinity [48], [50], [52], [53] and cold [45], [54] stresses. However, until now these responses have been seen only in heterologous systems. Transgenic *Arabidopsis* plants expressing ASR proteins from other plant species [42], [54] demonstrated increased tolerance to abiotic stresses. *Arabidopsis* plants do not encode ASR proteins. Ectopic expression of tomato *ASR1* in *Arabidopsis* was shown to affect the plant’s response to ABA, glucose and tolerance to abiotic stress via competition for DNA binding with the transcription factor ABI4 [55]. Thus, expression studies in heterologous organisms that do not naturally have the studied gene(s) should be analyzed with caution, especially for regulatory proteins, as results may not directly reflect the biological role of the analyzed gene. In addition, *ASR* gene involvement in carbohydrate signaling, sugar trafficking and metabolism has been shown [14], [24], [56], [57], as has its influence on the biogenesis of branched-chain amino acids [58].

In tomato, the best-studied member of the *ASR* family is *ASR1*, followed by *ASR2*. Information on *ASR3*, *ASR4* and *ASR5* is scarce. Although a few studies have compared the expression of some members of the tomato *ASR* gene family [59], [60], results are from northern blot, semi-quantitative PCR, or histological staining studies analyzing two or three of the family’s genes, under rather restricted conditions. In this work, we revisited the tomato *ASR* gene family using the highly accurate quantitative RT-PCR technology to determine the expression patterns of its members in vegetative and reproductive organs. Because of their high sequence homology, we could not design gene-specific primers for *ASR3* and *ASR5*, we determined the summed expression of these genes (*ASR3/5*). We found that *ASR1* and *ASR4* are highly expressed in vegetative tissues of nonstressed plants, whereas the steady-state levels of *ASR2* and *ASR3/5* transcripts are two to three orders of magnitude lower. *ASR1* was the major member expressed in roots, stems, stamens, pistils and fruits. *ASR4* accounted for more than two-thirds of total *ASR* transcripts in leaves, shoot vegetative meristem, sepals and petals. Both of these genes were induced by ABA, osmotic stress and salt stress. Tomato plants overexpressing *ASR1* (*ASR1*-OE) survived better under water stress than wild-type (WT) plants, where *ASR1*-antisense (*ASR1*-AS) plants had slightly lower survival rates than the WT.

## Materials and Methods

### Plant material and growth conditions

#### Generation and selection of transgenic lines

The 348-bp coding region of the tomato *ASR1* gene (GenBank U86130.1) was cloned in sense or antisense orientation into the multiple cloning site of the vector [61] between the *Cauliflower Mosaic Virus* (*CaMV*) 35S promoter and the *octopine synthase* (*ocs*) terminator. Tomato plants (*Solanum esculentum* cv. Moneymaker) were transformed by *Agrobacterium* as previously described [62]. Emerging shoots were excised and selected on Murashige and Skoog (MS) medium containing kanamycin (100 mg/l), and then transferred to the greenhouse for selection by qPCR in T1 plants. Transgenic plants were numbered XX-YY, where XX represents an independent transformation event, and YY represents a sub-line of the founder of T2 generation seeds. Plants were self-pollinated and seeds were collected. T3 generation plants were used in this study. Western blot analyses showed that the *ASR1*-OE lines have higher levels of *ASR1* than WT plants (Figure S1 in File S1).

#### Plant growth

Plants were grown in pots in the greenhouse or hydroponically in the growth room in aerated half-strength Hoagland mineral solution at 28°C and 70% relative humidity, under a diurnal cycle of 18 h light, or in the greenhouse at an average temperature of 28°C, and >50% relative humidity. Seeds were germinated in water-soaked vermiculite. Ten-day-old seedlings were transferred to aerated containers with half-strength Hoagland mineral solution [63], or to pots containing equal volumes of planting mix and vermiculite. Hydroponic growth medium was replaced 1 week after transfer and every 3–4 days thereafter.

#### Water-stress tolerance and survival assays

Seedlings were grown in pots under optimal conditions for 3 weeks. Water was then withheld for 22 days, followed by rewatering. Plant survival was scored 17 days later. At least 20 plants from each line were used. Plants were grown in random order and pot location was changed every few days.

### NaCl, polyethylene glycol (PEG) and ABA treatment

WT seedlings were germinated on vermiculite and transferred to aerated 0.5X Hoagland’s solution as described above. After 1 week acclimation, plants were transferred to fresh mineral solution containing, where indicated, 40 µM ABA, 0.2 M NaCl, or 8% (w/v) PEG 8000. Plants were harvested 24 h after treatments.

### mRNA level assay

Total RNA was extracted from the indicated plant tissues using EZ-RNA (Biological Industries, Israel) according to the manufacturer’s protocol. This protocol uses improved RNA-extraction methods as described by Chomczynski and Sacci [64]. RNA quality integrity was checked spectrally (at 230 nm, 260 nm and 280 nm) and by running samples on denaturing agarose gels electrophoresis. Relative steady-state transcript levels were assayed by RT-qPCR as described previously [50], [55], [65], [66]. cDNA was synthesized from DNase-treated RNA using high-capacity cDNA reverse transcription kit (Applied Biosystems). Primers were designed by Primer-Express software Vers. 2.0 (Applied Biosystems). When possible, one of the primers in each set was anchored at an exon–exon border to reduce possible amplification from contaminating genomic DNA. All amplicon lengths were between 75 and 90 bp. Primer sequences are presented in Table S1 in File S1. Transcript levels were assayed using the 7300 Real-Time PCR System (Applied Biosystems), with 18S rRNA as the internal standard. PCR efficiency was close to 100%. RNA relative quantification analyses were performed using 7300 System SDS software (Applied Biosystems). The list of primers used is shown in Table S1 in File S1. The data represent the mean ± SE of *n* = 3 independent experiments. Each data point was determined in triplicates in each of the three biological replicates and presented as mean ± SE. Data presented in a single graph were carried in a single run. Differences between groups were analyzed by Tukey’s HSD post-hoc test (P≥0.05).

## Results and Discussion

### Relative expression levels of the tomato *ASR* genes

**Figure 1:**
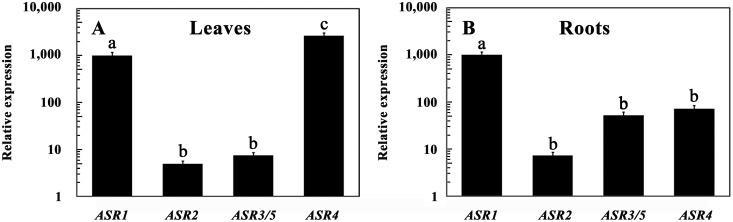
Relative expression of the tomato *ASR* genes. Steady-state levels of the indicated genes were determined in leaves and roots of 10-day-old hydroponically grown tomato seedlings. Expression of *ASR1* in each tissue was defined as 1000. Data shown are average ± SE. Bars with different letters represent statistically different values by Tukey’s HSD post-hoc test (P≤0.05).

Relative steady-state levels of the members of the *ASR* gene family were determined in leaves and roots of hydroponically grown seedlings. In general, *ASR1* and *ASR4* were the most highly expressed genes in this family (Figure 1). In young leaves, *ASR4* levels were 2.6 times higher than those of *ASR1*, whereas transcript levels of *ASR2* and *ASR3/5* were more than two orders of magnitude lower. In tomato roots, *ASR1* was the most abundantly expressed gene, whereas transcript levels of *ASR3*/5 and *ASR4* were approximately one order of magnitude lower than that of *ASR1*, and that of *ASR2* two orders of magnitude lower than the steady-state levels of *ASR1*. These results are in agreement with Frankel et al. [30] who reported that *ASR2* and *ASR3/5* transcripts could not be detected in tomato leaves by northern blot analysis. On the other hand, previous studies from the same laboratory reported that *ASR2* transcripts are highly abundant in roots of stressed tomato plants [60] and that the *ASR2* promoter can drive transcription in both tomato and other Solanaceae plant cells [60], [67]. RNA Seq also suggest that *ASR1* and *ASR4* transcripts in tomato leaves and fruits are relatively abundant, whereas transcripts from *ASR2*, *ASR3* and *ASR5* are hardly found [31].

(data presented at the tomato Sol Genomic Network database (http://solgenomics.net)).

### Expression of tomato *ASR* genes in vegetative tissues

**Figure 2:**
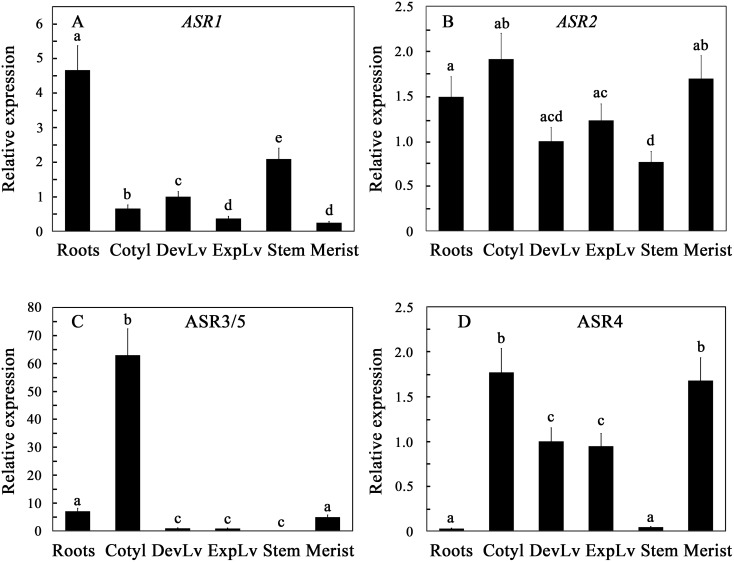
Expression of the tomato *ASR* genes in vegetative tissues. Steady-state levels of the indicated genes were determined in hydroponically grown tomato plants at the 8-true-leaf stage. The expression levels were normalized for each of the genes to their expression in young developing leaves, defined as 1. Cotyl, cotyledons; DevLv, developing leaves; ExpLv, fully expanded leaves; Merist, shoot meristem. Data shown are average ± SE. Bars with different letters represent statistically different values by Tukey’s HSD post-hoc test (P≤0.05).

Expression of tomato *ASR* genes was determined in vegetative tissues of hydroponically grown tomato (Figure 2). Transcript levels of each of the genes were normalized to the expression of the same gene in developing leaves. This reference tissue was selected since it is present in all plant ages used in the study. Highest levels of *ASR*1 transcript were found in roots and stems (ca. 4.5 and 2 times that in the leaves, respectively). *ASR1* transcript levels decreased with leaf development (Figure 2A), in agreement with Amitai-Zeigerson et al. [68]. *ASR1* expression in stems and roots suggests its role in the plant’s vascular system [59]. *ASR2* transcript levels were only slightly different in vegetative organs (Figure 2B), and its steady-state levels in all vegetative organs were marginal (see Figure 1). *ASR3/5* levels were highest in the cotyledons (Figure 2C), reaching transcript levels in the same order of magnitude of that of *ASR1* in cotyledons, suggesting that *ASR3* and/or *ASR5* my play a role in advanced stages of seed development. ASR proteins were detected immunologically in tomato seeds using anti-ASR1 antiserum [37]. Since there is high amino acid conservation between the different members of the tomato ASR proteins, is it likely that this antibody crossreacts with other ASR proteins such as ASR3 and ASR5. *ASR4* was expressed mainly in leaves, cotyledons and meristem, with relatively low expression rates in roots and stems (Figure 2D). After normalization of the relative expression of each gene in young leaf tissues (Figure 2A), *ASR1* seemed to be the main expressed gene in the roots and stems. On the other hand, *ASR4* was the highest expressed *ASR* gene in cotyledons, and young and fully expanded leaves. *ASR1* accounted for up to one quarter of of *ASR* gene family expression in these tissues-. Expression of *ASR2* and *ASR3/5* in leaves was negligible. Interestingly, and Our results indicate that *ASR1* is the primarily expressed gene in tomato roots, stems and fruits, whereas both *ASR1 and ASR4* are both expressed in cotyledons, leaves and meristems, where *ASR4* levels exceed that of *ASR1*. Transcript levels of *ASR2* and *ASR3/5* were marginal in vegetative tissues, with the exception of *ASR3/5* in cotelydons. Our analyzes determine averaged transcript levels in the entire organ, rather than cell specific expression. Thus, it will be interesting to find out if in these organs, *ASR1* and *ASR4* coexpress in the same cells, or in different cell types.

### Expression of the tomato ASR genes in reproductive tissues

**Figure 3:**
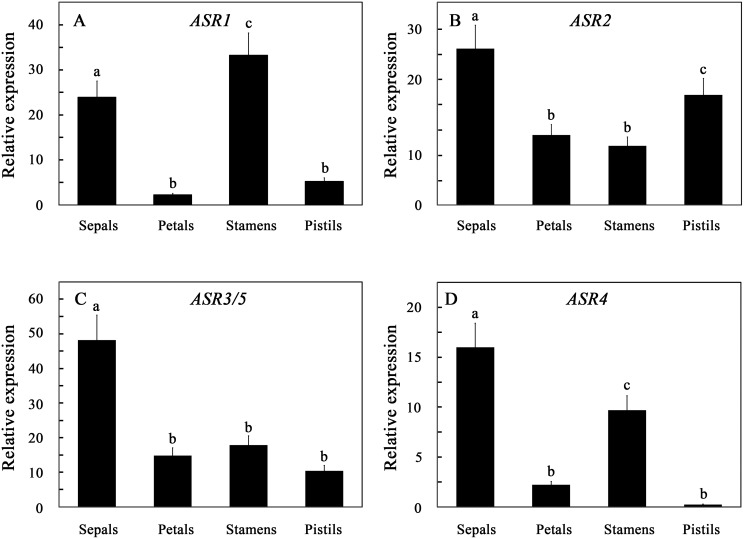
Expression of the tomato *ASR* genes in flower organs. Flowers and developing leaves were harvested from greenhouse-grown tomato plants. Flowers were dissected, and steady-state levels of the indicated genes were determined. The expression levels were normalized for each of the genes to their expression in young developing leaves, defined as 1. Data shown are average ± SE. Bars with different letters represent statistically different values by Tukey’s HSD post-hoc test (P≤0.05).

The *ASR* gene family showed differential expression in flower organs. Although *ASR2* and *ASR3/5* expression varied in the different flower organs (Figure 3B, C), their expression levels can be estimated to be two order of magnitude order lower than that of *ASR1* and *ASR4* of *ASR1* and *ASR4* were highly expressed in the sepals and stamens, and to a lower extent in petals and pistils (Figure 3A, D), where *ASR4* estimated transcript levels were higher than *ASR1* in the sepals and petals. One the other hand, *ASR1* is the most highly expressed *ASR* gene in the stamen and pistils.

**Figure 4:**
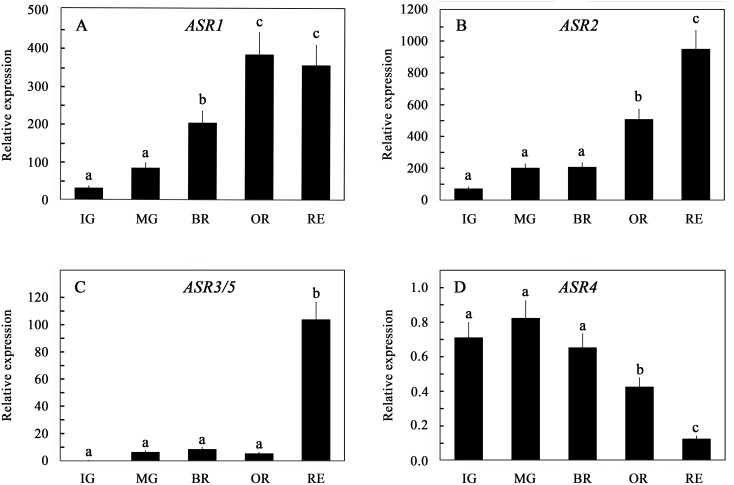
Expression of the *ASR* genes in fruit development. Fruits and developing leaves were harvested from greenhouse-grown tomato plants, and steady-state levels of the indicated genes were determined. The expression levels were normalized for each of the genes to their expression in young developing leaves, defined as 1. Fruit stages were defined according to [75]: IG, immature green; MG, mature green; BR, breaker; OR, orange; RE, red. Data shown are average ± SE. Bars with different letters represent statistically different values by Tukey’s HSD post-hoc test (P≤0.05).

Although *ASR1* expression increased during fruit development (Figure 4A), it was already higher than that in all other tissues in young immature green fruits. *ASR1* was essentially the main fruit-expressed *ASR* gene at all fruit developmental (Figure 4). Steady-state levels of *ASR2 and ASR3/5* transcripts in fruit tissues were also the highest measured in any plant tissue. Nevertheless, their levels in fruits are approximately two order of magnitude lower of that of *ASR1*. An increase in *ASR1* transcript levels in tomato fruit development and ripening is in agreement with Gilad et al. [4] and Iusem et al. [1], but not with Maskin et al. [60]. Increase in *ASR1* during tomato fruit ripeing also correlates with increase in its protein levels [20]. Increased steady state transcript levels during fruit ripening of *SlASR1* orthologous genes were reported in a number of plant species [21]–[23], [69], [70].

### Tomato ASR genes are differentially responsive to ABA and abiotic stress

**Figure 5:**
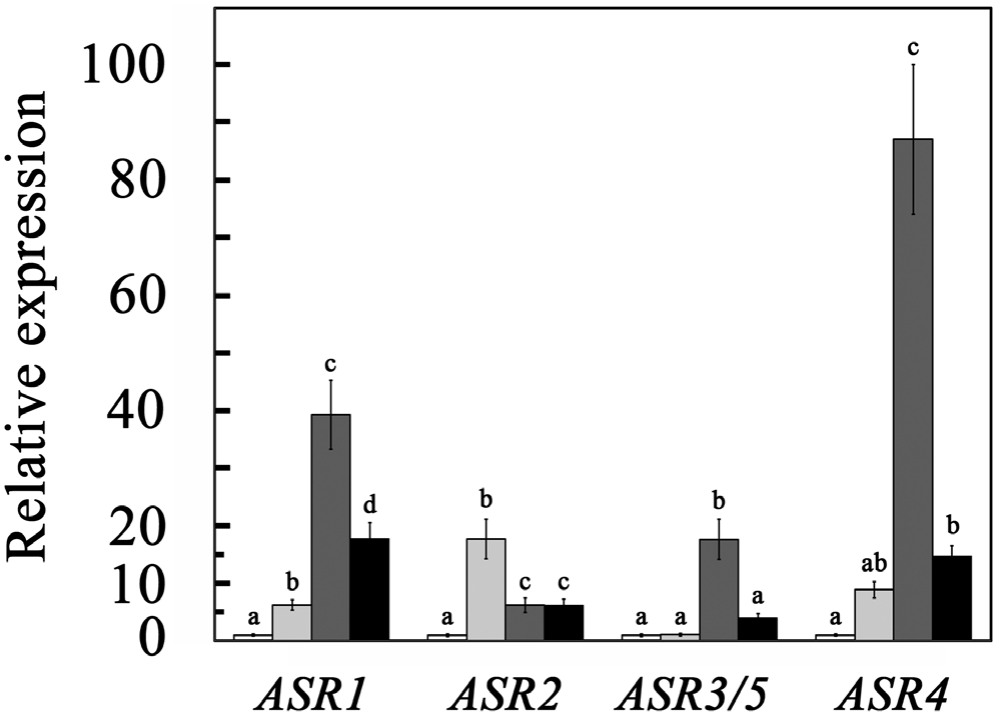
Effects of ABA, NaCl and PEG on the steady-state transcript levels of *ASR* genes. One-week-old hydroponically grown seedlings were transferred to fresh Hoagland solution containing: no addition (white bars), 40 µM ABA (light gray bars), 0.2 M NaCl (dark gray bars), or 8% PEG 8000 (black bars). Leaves were harvested 24 h later and expression levels were determined as described in Materials and Methods. Data were normalized for each of the genes to their expression in young developing leaves, defined as 1. Data shown are average ± SE. Expression of each gene in response to plant treatment was analyzed separately using Tukey’s HSD post-hoc test. Bars with different letters represent statistically different values by (P≤0.05).

Steady-state levels of *ASR1* and *ASR4* transcript increased following plant exposure to salt stress, osmotic stress (PEG) or to the hormone ABA (Figure 5). The relative induction levels by these treatments were rather similar for these two highly expressed *ASR* genes. On the other hand, the less expressed genes *ASR2* and *ASR3/5* showed different responses: *ASR2* responded most strongly to ABA treatment and to a lesser extent to salt or osmotic stress, whereas *ASR3*/5 was not affected by ABA and was relatively highly expressed after NaCl treatment, suggesting that they are induced by an ABA-independent pathway. A large number of *ASR* genes have been identified in different plant species based on their response to abiotic stresses such as water, salinity and osmotic stress [9], [10], [14], [71], [72]. Expression results for the tomato *ASR1* gene are in agreement with previous studies [4], [59], [60], [68]. In contrast, activity of the tomato *ASR2* promoter was enhanced by ABA when expressed in papaya and tobacco, but not in tomato or potato [67].

### Genetic manipulation of *ASR1* in tomato affects water-stress tolerance

**Figure 6:**
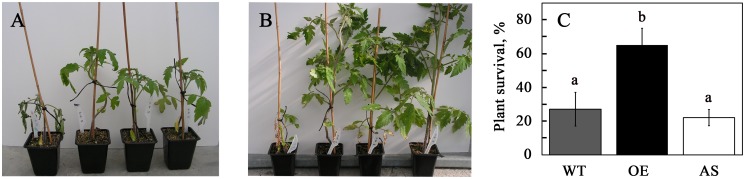
ASR1-overexpressing tomato plants have enhanced tolerance to water stress. Plants were grown in pots in the greenhouse using optimal irrigation for 17 days. Water was withheld for 22 days, following by rewatering for 17 days. Panels A and B show representative plants (left to right): wild type, *ASR1*-OE-31, *ASR1*-OE-12, *ASR1*-OE-16 after 22 days of dehydration (A) and 17 days of rewatering (B). Panel C, quantitative data of survival of three lines of *ASR1*-overexpressing (*ASR1*-OE) plants and four lines of *ASR1*-antisense (*ASR1*-AS) plants, measured at the end of the rewatering stage. Data shown are average ± SE. Bars with different letters represent statistically different values by Tukey’s HSD post-hoc test (P≤0.01).

Tomato plants overexpressing *CaMV 35S:ASR1* (*ASR1*-OE) or *CaMV 35S:Reverse ASR1* (*ASR1*-AS) were tested for tolerance to water and salt stresses. The modified plants did not perform significantly differently from WT plants when treated with NaCl (not shown). However, *ASR1*-OE plants survived water stress better than WT plants (Figure 6A and 6B). The response of *ASR1*-AS plants was highly variable: many lines showed hypersensitivity to water stress, whereas others showed no difference, or even better tolerance (Figure S2 in File S1). Averaging the recovery rates of all lines showed that *ASR1*-OE plants were significantly more tolerant to water stress than WT controls and *ASR1*-AS plants, whereas the latter were slightly more sensitive to lack of water than WT plants (Figure 6C). Transgenic plants overexpressing *ASR* genes have been reported to be more tolerant to abiotic stresses such as water/osmotic stress [42], [48]–[51], salinity stress [48], [50], [52], [53] and cold stress [44], [54]. However, most of those studies expressed the studied gene in a heterologous system [42], [48]–[50], [52]–[54]. In some of those studies, the biological species of the gene of origin and the transgenic plant belonged to the same botanical genus or family [24], [53], [56], but only a few studies have been performed within a single species [45], [51]. The biological relevance of studying the role of a regulatory protein on a genetic background that naturally lacks it has been questioned [55]: the phenotype of *Arabidopsis* plants expressing tomato *ASR1* was shown to result from competition for DNA binding between the ectopically expressed tomato gene and the Arabidopsis transcription factor ABI4, essentially resulting in an *abi4*-mutant-like phenotype [55]. Thus, expressing the studied gene on the same genetic background ([45], [51] and this study), or in closely related species [24], [53], [56], is more likely to shed light on the actual role of the studied gene and its protein product. The increased survival of transgenic tomato plants overexpressing tomato *ASR1* following water stress (Figure 6) is in agreement with reports on the expression of plant *ASR* genes on similar or different genetic backgrounds in response to stress [42], [48]–[51]. Results obtained using the *ASR1*-AS lines were more variable: lines ASR1-AS5-4 and ASR1-AS18-4 were significantly hypersensitive to water stress as compared to WT plants (Figure S2 in File S1), whereas other lines were not significantly different. On the other hand, no significant differences were found in the sensitivity of *ASR1*-OE and *ASR1*-AS plants to NaCl stress (not shown), most probably due to the relative tolerance of the tomato WT line. ASR proteins are localized in the cytosol and nucleus. In the latter organelle, they are associated with DNA [14], [39], [40], [48], [73]. Tomato and grape ASR proteins have been shown to bind specific DNA sequences [14], [53], suggesting that they regulate the expression of genes involved in abiotic stress responses or sugar metabolism [4], [14], [24], [54], [57], [74]. In addition, ASR proteins have been shown to possess protein-chaperone-like activity [38], [41], increasing protein stability. Thus, the increased water-stress tolerance of *ASR1*-OE plants most likely results from an increase in the transcription of ASR1-regulated genes and from ASR1 chaperon activity.

## Conclusions

Two of the five genes in the *ASR* gene family (*ASR1* and *ASR4*) are significantly expressed in tomato plants, whereas the expression levels of the other three member of the gene family are less pronounced. *ASR1* and *ASR4* encode 115- and 297-amino acid polypeptides, respectively, thus most likely encoding proteins whose activity may not be fully redundant. ASR1 is expressed in all plant tissues tested: it is most highly expressed in the stem, roots and reproductive organs–stamen, pistils and fruit at all developmental stages. *ASR4* is mainly expressed in photosynthetic organs, in in sepals and petals. The steady-state levels of *ASR1* and *ASR4* increased following salt stress, osmotic stress and treatment with ABA. *ASR2* expression is negligible in all tested tissues. *ASR3* and *ASR5*, being highly similar genes, were assayed together, with significant expression detected only in the cotyledons. These results suggest that *ASR2/3/5* may be very low expressing genes, or that their expression is limited to specific low abundant cells, thus resulting in low transcript activity when assayed in the whole tissue. Tomato plants overexpressing *ASR1* show increased tolerance to water stress, whereas *ASR1*-AS plants show a certain degree of hypersensitivity to water withholding. Our data suggest that *ASR1* and *ASR4* may be expressed in different cell types. The differential expression patterns of the tomato ASR gene family under non-stress and stress conditions may be used for genetic manipulation of tomato (as well as other crop plants) to affect vegetative and fruit parameters under non-stress and stress conditions.

## Supporting Information

File S1 **Western-blot analysis of** ***ASR1*** **-overexpressing tomato, response of individual transgenic lines to water stress, and list of primers used for RT-qPCR.**
(DOC)
