# Supplementary material for: Tomato ABSCISIC ACID STRESS RIPENING (ASR) Gene Family Revisited
Source: PLoS One. 2014 Oct 13;9(10):e107117. doi: 10.1371/journal.pone.0107117 (PMC4195575; doi:10.1371/journal.pone.0107117)
Supplement: Supplementary file 1 [file pone.0107117.s001.doc]

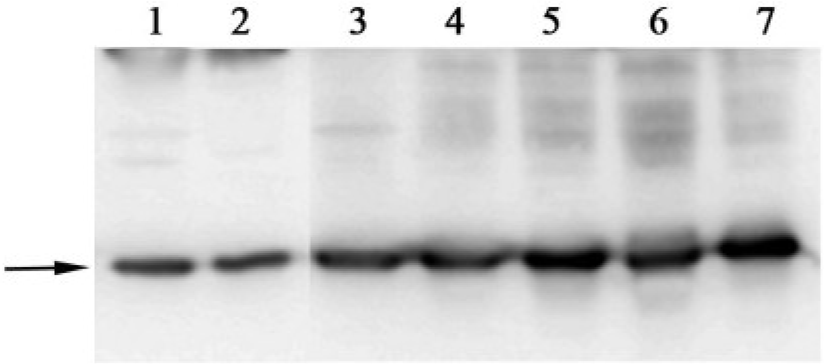

Figure S1. Western-blot analysis of *ASR1*-overexpressing (*ASR1*-OE) tomato.

Acid-soluble proteins were extracted from leaves of WT (lanes 1,2) and *ASR1*-OE plants (lanes 3–7). Proteins were resolved by SDS-PAGE, electroblotted onto nitrocellulose membrane, and probed with anti-ASR1 antibody. Arrow indicates the position of ASR1.

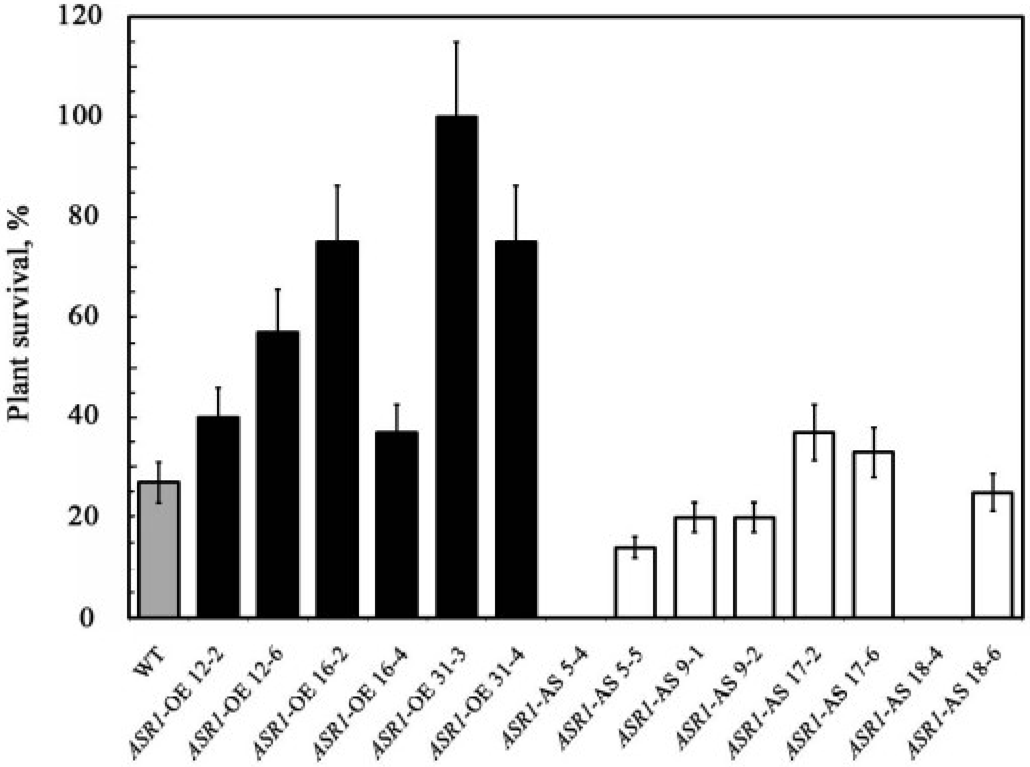

Figure S2. Survival rates of *ASR1*-overexpressing and *ASR1*-antisense plants following water stress. Plants from the indicated lines (at least 10 plants per line) were grown in pots in the greenhouse using optimal irrigation for 17 days. Water was withheld for 22 days, following by rewatering for 17 days. Gray bar, wild type; black bars, *ASR1*-overexpressors; white bars, *ASR1*-antisense. Data shown are average ± SE.

Table S1. Primers used for qRT-PCR.

Gene	Primer	Sequence (5'→3')
Sl*ASR1*	ASR1F	GGGACACCACCATCTCTTCTAAA
	ASR1R	CCAAATATGGAAATTCCACGAATAT
Sl*ASR2*	ASR2F	GACATTAATTTAAGAGAAGCAATACAATATGG
	ASR2R	GGTGGAACAAATGGTGATGGT
Sl*ASR3/5*	ASR3F	AAATTGTCTATCGACGTTTAATCAACA
	ASR3R	TGTGGTGGAACAAACGGTGA
Sl*ASR4*	ASR4F	GGTAATGAGGAAGGTGGCTATGG
	ASR4R	TGGTTCCACTATCATCATTCTCTTCA
Sl18SrRNA	18SF	GGAGTATGGTCGCAAGGCTG
	18SR	AGTCAAATTAAGCCGCAGGC
